# Molecular signature associated with cladribine treatment in patients with multiple sclerosis

**DOI:** 10.3389/fimmu.2023.1233546

**Published:** 2023-07-25

**Authors:** Nicolas Fissolo, Laura Calvo-Barreiro, Herena Eixarch, Ursula Boschert, Luisa M. Villar, Lucienne Costa-Frossard, Mireia Ferrer, Alex Sanchez, Eva Borràs, Eduard Sabidó, Carmen Espejo, Xavier Montalban, Manuel Comabella

**Affiliations:** ^1^Servei de Neurologia, Centre d’Esclerosi Múltiple de Catalunya (Cemcat) Institut de Recerca Vall d’Hebron (VHIR), Hospital Universitari Vall d’Hebron, Universitat Autònoma de Barcelona, Barcelona, Spain; ^2^Ares Trading SA, Eysins, Switzerland, an affiliate of Merck KGaA, Darmstadt, Germany; ^3^Department of Immunology, Multiple Sclerosis Unit, Hospital Ramon y Cajal, Instituto Ramón y Cajal de Investigación Sanitaria (IRYCIS), Madrid, Spain; ^4^Department of Neurology, Multiple Sclerosis Unit, Hospital Ramon y Cajal, Instituto Ramón y Cajal de Investigación Sanitaria (IRYCIS), Madrid, Spain; ^5^Statistics and Bioinformatics Unit, Vall d’Hebron Institut de Recerca (VHIR), Barcelona, Spain; ^6^Genetics, Microbiology and Statistics Department, Universitat de Barcelona, Barcelona, Spain; ^7^Proteomics Unit, Centre de Regulació Genòmica (CRG), Barcelona Institute of Science and Technology (BIST), Barcelona, Spain; ^8^Proteomics Unit, Universitat Pompeu Fabra, Barcelona, Spain

**Keywords:** multiple sclerosis, cladribine, biomarkers, omics, bioinformatics

## Abstract

**Introduction:**

Little is known about the molecular profiling associated with the effect of cladribine in patients with multiple sclerosis (MS). Here, we aimed first to characterize the transcriptomic and proteomic profiles induced by cladribine in blood cells, and second to identify potential treatment response biomarkers to cladribine in patients with MS.

**Methods:**

Gene, protein and microRNA (miRNA) expression profiles were determined by microarrays (genes, miRNAs) and mass spectrometry (proteins) in peripheral blood mononuclear cells (PBMCs) from MS patients after *in vitro* treatment with cladribine in its active and inactive forms. Two bioinformatics approaches to integrate the three obtained datasets were applied: (i) a multiomics discriminant analysis (DIABLO - Data Integration Analysis for Biomarker discovery using Latent variable approaches for Omics studies); and (ii) a multi-stage integration of features selected in differential expression analysis on each dataset and then merged. Selected molecules from the *in vitro* study were quantified by qPCR *ex vivo* in PBMCs from MS patients receiving cladribine.

**Results:**

PBMCs treated *in vitro* with cladribine were characterized by a major downregulation of gene, protein, and miRNA expression compared with the untreated cells. An intermediate pattern between the cladribine-treated and untreated conditions was observed in PBMCs treated with cladribine in its inactive form. The differential expression analysis of each dataset led to the identification of four genes and their encoded proteins, and twenty-two miRNAs regulating their expression, that were associated with cladribine treatment. Two of these genes (PPIF and NHLRC2), and three miRNAs (miR-21-5p, miR-30b-5p, and miR-30e-5p) were validated *ex vivo* in MS patients treated with cladribine.

**Discussion:**

By using a combination of omics data and bioinformatics approaches we were able to identify a multiomics molecular profile induced by cladribine *in vitro* in PBMCs. We also identified a number of biomarkers that were validated *ex vivo* in PBMCs from patients with MS treated with cladribine that have the potential to become treatment response biomarkers to this drug.

## 1 Introduction

Cladribine (2-chlorodeoxyadenosine) is a synthetic deoxyadenosine analogue originally developed as chemotherapeutic molecule for the treatment of haematological malignancies (1), that has shown efficacy in relapsing-remitting multiple sclerosis (RRMS) patients (2, 3). Cladribine is a prodrug that gets into the cells via purine nucleoside transporters. Once inside, the molecule is converted into the active triphosphorylated deoxyadenosine by means of phosphorylation performed by the deoxycytidine kinase (DCK) (4, 5).

The main mechanism of action defined for cladribine is the induction of lymphopenia by apoptosis of T and B cells (6, 7). However, other immune regulatory functions beyond cytotoxicity of T and B lymphocytes have also been postulated, such as impairment of cell proliferation and activation of different cell subpopulations in peripheral blood mononuclear cells (PBMCs) (8–10).

These immunomodulatory effects induced by cladribine have been described as dependent on its activation by DCK. Nevertheless, cladribine could also trigger other immunomodulatory pathways in its inactive form, by means of agonist of adenosine receptors, mostly over A1 and A2A receptors (11). In this line, adenosine receptors are known to play an important role modulating inflammatory processes, inhibiting tissue damage produced by inflammation, and inducing anti-inflammatory responses (12). In the central nervous system, adenosine receptors have been reported to play important functions both in physiological and pathophysiological conditions (13).

Although the cellular immunomodulatory effects of cladribine on PBMCs have long been extensively studied and described (8, 14), the molecular profiling associated with cladribine treatment has not yet been investigated using omics technologies. In this context, the characterization of the transcriptional and proteomic changes induced by cladribine will contribute not only to a better understanding of its mechanism of action, but also to the identification of potential treatment response biomarkers in RRMS patients receiving cladribine.

Bearing this in mind, in the present study we assessed the gene, miRNA, and protein expression profiling induced by cladribine *in vitro* in PBMCs from MS patients both in DCK-dependent and -independent conditions. By means of different bioinformatics approaches, we then integrated the three datasets and characterized the multiomics biomarker signature associated with cladribine. Finally, *in vitro* findings were validated *ex vivo* in MS patients undergoing treatment with cladribine.

## 2 Materials and methods

### 2.1 Ethics

Informed consent was obtained from all participants. The study was approved by the Vall d’Hebron Hospital, Barcelona [EPA(AG)57/2013 (3834)] and the Hospital Ramón y Cajal, Madrid.

### 2.2 Subjects

The effect of cladribine on gene, miRNA and protein expression was explored *in vitro* in PBMCs obtained from ten RRMS patients. Seven patients were treatment naïve and three patients received in the past teriflunomide, glatiramer acetate and interferon-beta but were untreated for the last six months before blood collection. **Table 1** summarizes the main demographic and baseline clinical characteristics of patients included in the study.

**Table 1:** Demographic and baseline clinical characteristics of MS patients included in the study.

Baseline characteristics	*In vitro*	*Ex vivo*
n	10	5
Age (years)	35.3 (7.4)	35.3 (8.8)
Female/male (% women)	7/3 (70)	3/2 (60)
Duration of disease (years)	5.7 (2.9)	4.2 (2.1)
EDSS^a^	2.7 (1.6-3.0)	2.2 (1.5-2.5)

Data are expressed as mean (standard deviation) unless otherwise stated. ^a^Data are expressed as median (interquartile range). EDSS: Expanded Disability Status Scale.

### 2.3 Media and reagents

Human PBMCs were isolated using standard Ficoll gradient centrifugation. Cells were cultured in complete medium made with RPMI 1640 growth medium (Life Technologies, CA, USA) supplemented with 1% penicillin, 1% streptomycin, 1% glutamine, and 5% fetal calf serum (FCS; Gibco, Carlsbad, CA. USA). Cladribine (Merck, Darmstadt, Germany) and deoxycytidine (Sigma-Aldrich, St. Louis, MO, USA) were dissolved in DMSO and water respectively and further diluted to the appropriate concentration in complete medium

### 2.4 *In vitro* stimulation and treatment of PBMCs with cladribine

Isolated PBMCs were cultured in complete medium at 5% CO_2_, 37°C, and >95% humidity on 24-well plates, at a concentration of 1x10^6^ cells/ml. Cells were stimulated with 10 ng/ml phorbol 12-myristate 13-acetate (PMA) plus 500 ng/ml ionomycin (Sigma-Aldrich) and simultaneously treated with 0.1 μM cladribine in the presence or absence of 250 μM deoxycytidine (added 30 min prior to the addition of cladribine) for 4h. After *in vitro* treatment with cladribine, 2.5x10^6^ PBMCs were harvested in separated tubes (for RNA and protein extraction), pelleted (10 min, 400 × g) and frozen as dry pellets on dry ice, and stored at −80°C until all samples were collected. In this context, the following experimental groups were considered in the study: no cladribine, cladribine, cladribine plus deoxycytidine, and only deoxycytidine.

These *in vitro* culture conditions were based on previous studies performed by our group which demonstrated that untreated PBMCs needed to be stimulated first in order to induce activation and cell proliferation (8). The activation status after PBMC culture was tested by flow cytometry (by means of CD69 expression), and only experiments in which PBMCs showed a percentage of activation higher than 90% compared with the unstimulated condition were used to obtain RNA and protein for further purposes (**Figure S1**). The concentration of cladribine used in the *in vitro* experiments (0.1 μM) corresponds to the mean concentration present in plasma of patients with MS under cladribine treatment (15) The use of deoxycytidine, a molecule that competes with cladribine for the phosphorylation via DCK, will allow to investigate whether the inhibition of cladribine activation by DCK produces changes in expression patterns.

### 2.5 Analysis of gene and miRNA expression by microarrays

Total RNA was extracted from PBMC pellets with the miRNeasy Mini Kit Qiagen (Qiagen, Basel, Switzerland) following the manufacturer’s protocol. RNA quantity and quality were determined with a Bioanalyzer 2100 (Agilent Technologies, Santa Clara, CA, USA). Gene expression was assessed using the Clariom™ S array (Affymetrix, Santa Clara, CA, USA), and miRNA expression was determined with the GeneChip^®^ miRNA 4.0 Array (Affymetrix). Data processing and normalization were carried out using the R statistical language (https://www.r-project.org/) (version 3.6.3) and the libraries created for microarray analysis in the Bioconductor Project (16). In order to get normalized expression values (log2 scale), raw expression values obtained directly from.CEL files were processed using the Robust Multiarray Average (RMA) algorithm in oligo package (17). Linear models for microarray data (LIMMA) R package was used to identify differentially expressed genes and miRNAs between the different groups (18). Genes and miRNAs showing a differential expression by two-sample t-test (p-value <0.05), were considered significant.

### 2.6 Mass spectrometry sample preparation

PBMC pellets were lysed in RIPA buffer (1% sodium deoxycholate, 1% Triton X-100, 0.15 M NaCl, 0.1% SDS, 0.01 M sodium phosphate, pH 7.2) supplemented with 1 mM Na_3_VO_4_, 0.5 mM DTT, and protease inhibitor mixture. Protein concentration was determined using Pierce BCA Protein Assay Kit (Thermo-Fisher Scientific, San Jose, CA, USA) as recommended by the manufacturer. Then, samples (10ug) were precipitated with cold acetone and the protein pellets were dissolved in 6M Urea/200mM ammonium bicarbonate. Samples were reduced and alkylated (dithiothreitol and iodoacetamide) prior to tryptic digestion (LysC and Trypsin) and the resulting peptide mixtures were acidified with formic acid and desalted with a MicroSpin C18 column (The Nest Group, Inc; Southborough, MA).

### 2.7 Chromatographic and mass spectrometric analysis

Samples were analyzed by mass spectrometry (Orbitrap Fusion Lumos) coupled to an EASY-nLC 1200 (Thermo-Fisher Scientific). Peptides were separated by reversed-phase chromatography using C18 columns of 50 cm length and 75 μm inner diameter in chromatographic gradients of 90 minutes (water, acetonitrile, 1% formic acid) and a flow rate of 300 nl/min. The mass spectrometer was operated in data-dependent acquisition mode, MS1 scans were acquired in a mass range of 350-1,400 m/z and detected in the Orbitrap at 120.000 resolution. The most intense precursor ions selected by the instrument (“Top Speed” algorithm and 60 seconds dynamic exclusion) were then fragmented by high-energy collision dissociation (HCD) at 28% normalized collision energy and detected in the ion trap. To control the instrument performance (19) and avoid for carryover, digested bovine serum albumin was run between each sample.

### 2.8 Mass spectrometry data analysis

Mass spectrometry data was analyzed by Proteome Discoverer v2.0 (Thermo-Fisher Scientific) and Mascot search engine v2.6 (Matrix Science) (20) using the Swiss-Prot human database (February 2020) plus a list of common contaminants and all the corresponding decoy entries (21). Mass tolerances were set to 7 ppm and 0.5 Da for MS1 and MS2 respectively. Tryptic peptides with a maximum of three missed cleavages were permitted. Carbamidomethylation on cysteines was set as a fixed modification and the variable modifications of N-terminal protein acetylation and methionine oxidation were also considered. For peptide identification, we set a maximum of 5% false discovery rate.

Protein Abundances were estimated as the average of the three most abundant peptides and they were normalized by equalizing the median protein abundance in all samples. The raw proteomics data can be found in the repository PRIDE (22) with the identifier PXD040572.

### 2.9 Bioinformatics analysis

Data obtained from arrays of genes, miRNAs, and mass spectrometry were processed as described above to get the normalized, log-transformed expression values, and then analyzed using two different integrative bioinformatics approaches:

(i) DIABLO (Data Integration Analysis for Biomarker discovery using Latent variable approaches for Omics studies): This algorithm selected the most predictive or discriminative molecules in the datasets that helped to classify the samples (23). Before running this analysis, three types of parameters were tuned. (i) Matrix design: a minimal link of 0.1 between the datasets was used to maximize the discriminative power of the analysis. (ii) Number of components: was set on 2. (iii) Number of features to select on each dataset/component: based on a sPLS-DA (sparse Partial Least Squares-Discriminant Analysis) method, the optimal number of features to select on gene, protein and miRNAs was 40, 20, and 40, respectively. This approach will allow to have an overall multiomics picture of the most relevant molecular patterns associated with the different phenotypes.

(ii) Multi-stage: the individual omics databases are first analyzed separately and the results are then merged and compared (18). For the differential expression analysis, genes and proteins with a p-value <0.5, and miRNAs with a p-value <0.5 and absolute log2Fold-Change > 0.5 in the comparison between cladribine treated and the untreated conditions were selected for further analysis. This approach will allow to focus on individual candidate biomarkers functionally connected in the three datasets that are associated with the effect of cladribine.

The multiMiR Bioconductor’s package was employed to identify validated targets for the miRNAs differentially expressed between the treated and untreated conditions. Such analysis enables retrieval of validated miRNA-gene target interactions from several external databases (miRTarBase, miRecords, and TarBase databases) (24).

### 2.10 Selection of molecules for the bioinformatics analysis

Before integration of the three datasets (gene/protein/miRNA), a filter was applied in order to eliminate molecules that either did not differentiate from the rest in the same dataset or were not detected in most of the samples included in the study. In the gene expression dataset, a filtering step was performed based on a variance threshold of 65%, and transcripts with a low variability or without a valid Entrez ID annotation were removed from the analysis. For the miRNA dataset, probes belonging to other species (non-human), control probesets, and duplicates were removed. For the proteomic dataset, proteins with a confidence score < 20, as well as proteins detected in < 25% of the samples in each group were discarded (23).

### 2.11 Determination of gene and miRNA expression levels *ex vivo* by qPCR

Gene and miRNA expression levels were determined *ex vivo* by qPCR in PBMCs from five RRMS patients at baseline and after 3 and 12 months of cladribine treatment onset. Three patients were treatment naïve, and 2 patients were treated before with dimethyl fumarate and one with glatiramer acetate. A summary of demographics and clinical characteristics of the *ex vivo* cohort is given in **Table 1**. Total RNA was extracted from PBMCs as previously described for the microarray studies, following a reverse transcription into complementary DNA (cDNA) using the High capacity cDNA Archive kit (Applied BiosystemsTM, Foster City, CA, USA) for gene expression, and the TaqManTM Advanced miRNA cDNA synthesis kit (Applied Biosystems) for miRNA expression experiments. Gene expression levels of NIBAN2 (niban apoptosis regulator 2), NHLRC2 (NHL repeat containing 2), PPIF (Cyclophilin D), and JUN (Transcription factor Jun) transcripts were determined with TaqMan gene expression assays (Hs00382858_m1, Hs00612275_m1, Hs00194847_m1, and Hs01103582_s1, respectively) and TaqMan Fast Advanced Master Mix (Applied Biosystems). The obtained values were normalized according to the level of expression of the housekeeping gene glyceraldehyde-3-phosphate dehydrogenase (GAPDH). The miRNAs expression levels of miR-30e-5p, miR-766-3p, miR-21-5p, miR-30b-5p, and miR-484 were determined with TaqMan Advanced miRNA assays (479235_mir, 478342_mir, 477975_mir, 478007_mir, 478308_mir, respectively) and the TaqMan Fast Advanced Master Mix (Applied Biosystems), according the manufacturer. Assay for miR-191-5p (477952_mir) was used as a reference miRNA for normalization purposes. Both assays (gene and miRNA) were run on the ABI PRISM^®^ 7900HT system (Applied Biosystems) Data was analyzed with the 2-ΔCT method (25). Results were expressed as fold change in gene and miRNA expression at 3 and 12 months of cladribine treatment compared with the baseline (calibrators).

### 2.12 Statistical analysis

Statistical analysis was conducted using SPSS (Version 20) software (SPSS Inc, Chi-cago, IL, USA) for MS-Windows and GraphPad Prism 6.0 software (GraphPad Prism Inc, La Jolla, CA, USA). Expression values are represented using boxplots. Adjusted p-values for omics data were obtained using the Benjamini & Hochberg method. One-way ANOVA with repeated measures following a Dunnett multiple comparisons test was used, taking the control condition (baseline) as reference.

### 2.13 Data availability

The data presented in this study are available on request from the corresponding author.

## 3 Results

### 3.1 Integrative omics analysis reveals a general downregulation expression signature associated with cladribine effect

**Figure 1:**
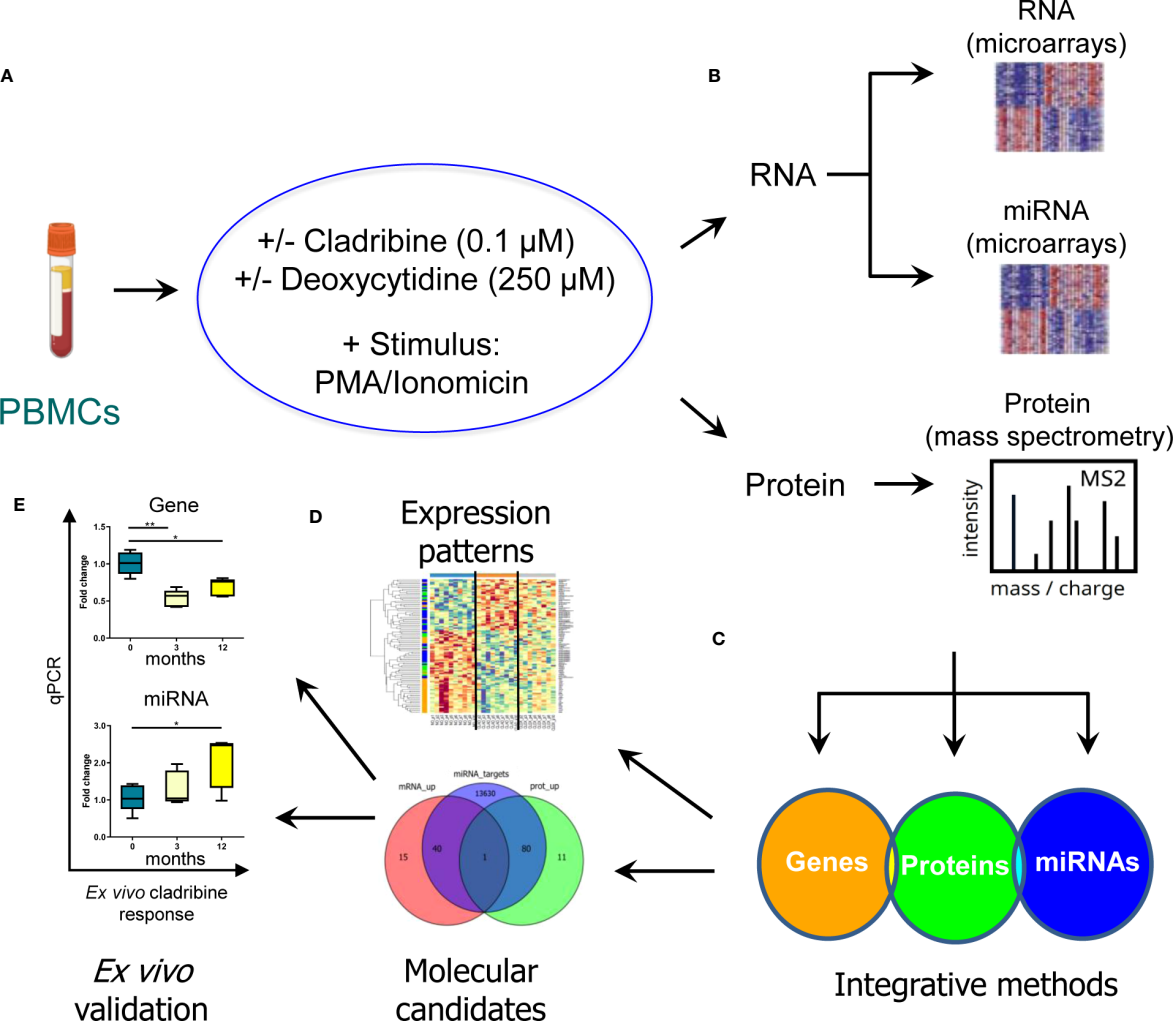
Schematic representation of the strategy used to characterize the molecular effect of cladribine. In order to evaluate the molecular profiling associated with the *in vitro* effect of cladribine, PBMCs obtained from MS patients were first stimulated with PMA/ionomicin and then treated with cladribine in the presence or absence of an excess of deoxycytidine, a competitive substrate for DCK-dependent phosphorylation **(A)**. Subsequently, the transcriptomic and proteomic profile was determined by means of gene and miRNA expression microarrays, and mass spectrometry in the different experimental conditions **(B)**. Aiming to identify a multiomics biomarker signature associated with cladribine treatment, we applied two different bioinformatics approaches to integrate the three generated databases (gene, miRNAs, and proteins) **(C)**. These analyses allowed us, on the one hand, to characterize a multiomics expression pattern associated with each experimental condition and, on the other hand, to identify potential candidate biomarkers associated with cladribine exposure **(D)**. Finally, individual candidates were validated *ex vivo* in MS patients receiving treatment with cladribine **(E)**. *p-value <0.05, **p-value <0.01.

A schematic overview summarizing the main steps of the study design and analysis is represented in **Figure 1**.

**Figure 2:**
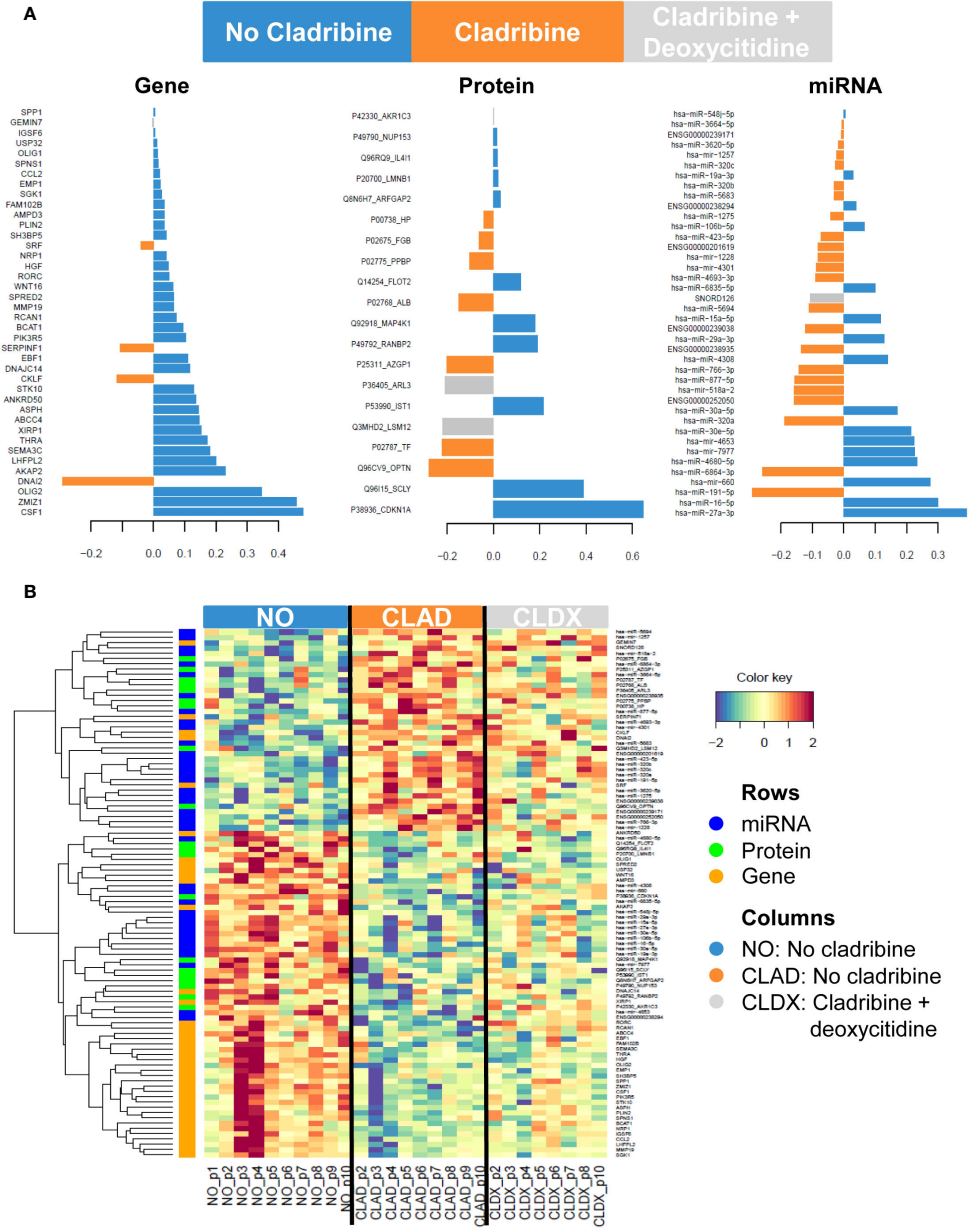
Gene, protein and miRNA expression profiles associated with cladribine effect. **(A)** Loading plots showing the contribution of the most discriminative features in each dataset comparing the three phenotypes: no cladribine, cladribine, and cladribine plus deoxycytidine. The length of the bar indicates the weight of the contribution of each feature in each group. **(B)** Clustered heatmap showing the gene, protein and miRNA expression profiles for each sample. Rows represent genes, proteins and miRNAs; columns represent profiled samples. The relative expression of each probe is depicted according to the color scale. Red indicates upregulation; blue indicates downregulation. The ID of the selected features represents the Gene Symbol for genes, the combined Uniprot accession number + Gene Symbol for proteins or the miRBase symbol for miRNA data. NO, no cladribine; CLAD, cladribine; CLDX, cladribine + deoxycytidine.

To reveal the molecular expression pattern associated with cladribine treatment, we applied the DIABLO bioinformatics approach which identified a multiomics molecular signature, by finding co-expression between the molecules from the different datasets measured in the same samples that best explained the study phenotypes: no cladribine, cladribine, and cladribine plus deoxycytidine. The only deoxycytidine condition was not included in the analysis, as this group showed a similar expression pattern to the no cladribine condition (data not shown). **Figure 2A** shows the molecules in the three databases with the highest differences between the groups. **Figure 2B** shows the unsupervised hierarchical clustering analysis performed with the features presenting the highest differences at the gene, protein, and miRNA levels. Overall, cladribine induced an expression signature characterized by general downregulation of the three different types of molecules, most noticeable at the gene expression level compared with the untreated condition. Interestingly, the cladribine plus deoxycytidine condition was characterized by an intermediate expression signature between the cladribine treated and untreated conditions at the three molecular levels, genes, proteins and miRNA. The lists of features included in the clustered heatmap and loading plots are shown in **Supplementary Table S1** (for genes), **Supplementary Table S2** (for proteins), and **Supplementary Table S3** (for miRNAs).

### 3.2 Individual database analysis identifies common dysregulated genes and proteins in PBMCs after cladribine treatment

**Figure 3:**
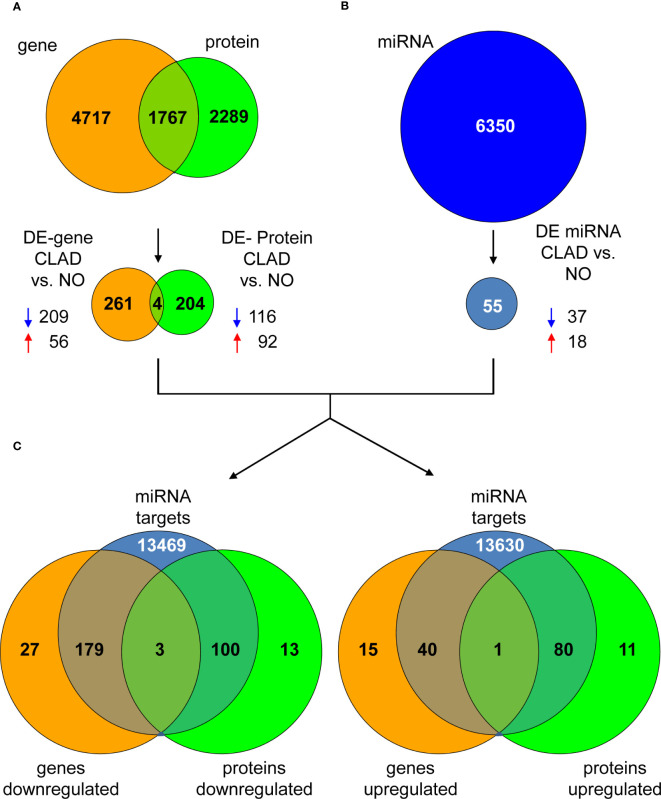
Dysregulated genes, proteins and miRNAs in cladribine treated vs. untreated PBMCs. **(A)** The Venn diagram shows all identified genes and proteins, as well as the common overlapping features. A total of 265 genes (209 down- and 56 up-regulated) and 208 proteins (116 down- and 92 upregulated) were differentially expressed between the cladribine treated and untreated conditions, of which 4 molecules were common in both datasets. **(B)** A total of 6,350 miRNAs were identified by microarrays, of which 55 were differentially expressed (37 down- and 18 upregulated) between the treated and untreated conditions. **(C)** Venn diagrams showing the overlapping of the 4 common differentially expressed genes and proteins previously identified, with all validated interactions found for the 55 differentially expressed miRNAs in the same comparison (treated versus untreated conditions). Overlapping graphs for the downregulated genes and protein are shown on the left and upregulated molecules on the right. DE, differentially expressed. CLAD, PBMCs treated with cladribine. NO, untreated PBMCs. Blue and red arrows indicate down- and upregulation, respectively.

**Figure 4:**
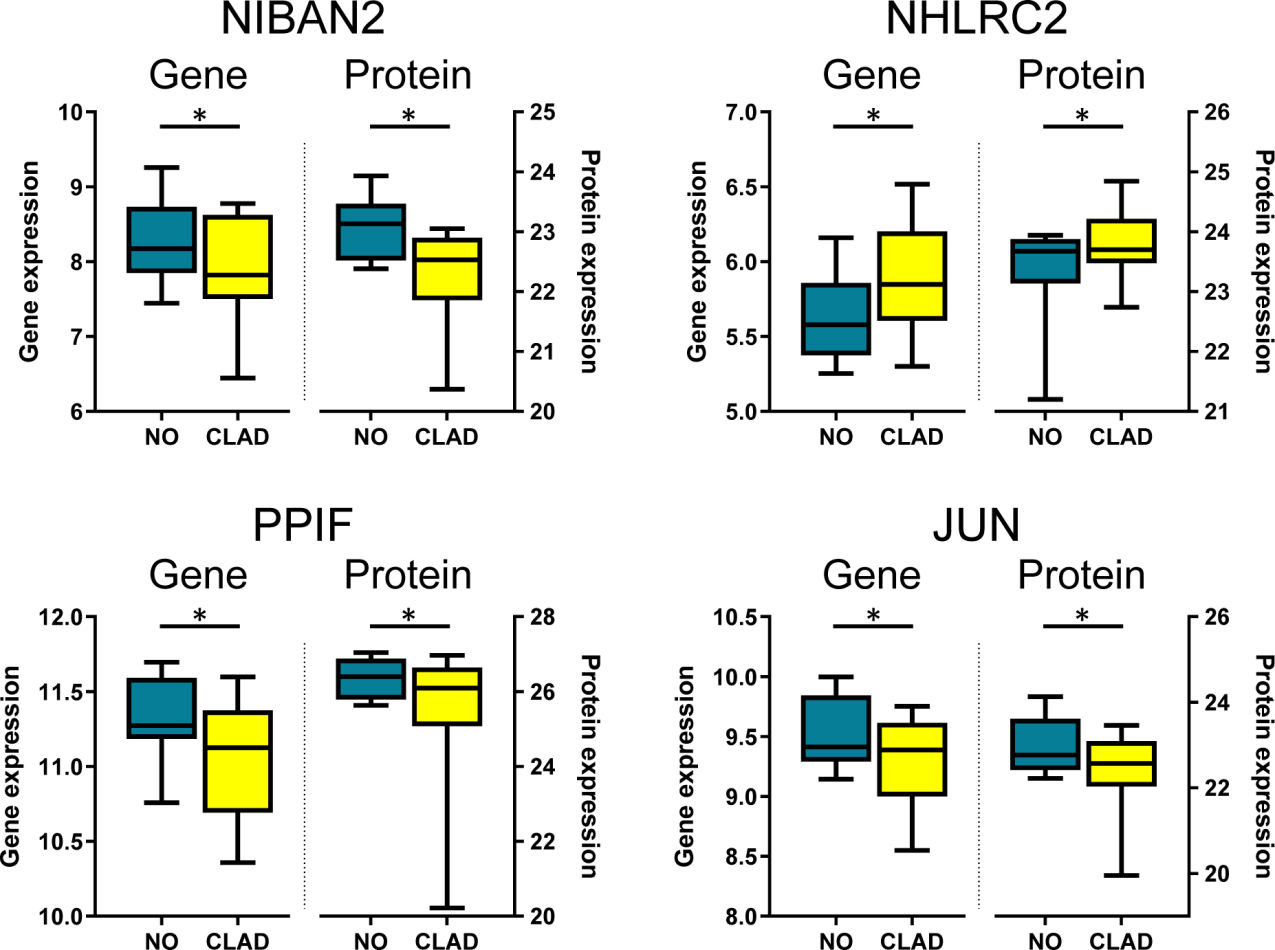
Candidate biomarkers associated with cladribine treatment showing differential expression both at the gene and protein levels. Boxplots depict gene and protein expression levels obtained with microarrays (genes) and mass spectrometry (proteins) of NIBAN2, NHLRC2, PPIF, and JUN in PBMCs treated *in vitro* with cladribine (CLAD). Results are compared with the untreated condition (NO). *Adjusted p-values. NIBAN2, niban apoptosis regulator 2. NHLRC2, NHL repeat containing 2. PPIFm Cyclophilin D. JUN, Transcription factor Jun.

In order to identify individual biomarkers candidates associated with cladribine treatment, we applied a multi-stage approach to analyze the common genes and proteins differentially expressed in the comparison between the cladribine treated and untreated conditions. The gene expression microarray and mass spectrometry analyses in PBMCs allowed to identified, after applying the corresponding filtering steps, 6,484 genes and 4,056 proteins respectively, of which 1,767 molecules were found in both studies (**Figure 3A**). Of these, 265 genes and 208 proteins were differentially expressed (p-values < 0.05) between the treated and untreated conditions. In line with the previous integrative analysis (DIABLO), cladribine induced a major downregulation of gene expression with 209 molecules downregulated vs. 56 upregulated (**Figure 3A**). Cladribine also reduced protein expression with 116 molecules downregulated and 92 upregulated after treatment (**Figure 3A**). Of note, four of these molecules, NIBAN2, NHLRC2, PPIF, and JUN, were common in both gene and protein datasets. As shown in **Figure 4**, gene and protein expression levels of NIBAN2, PPIF and JUN were significantly downregulated in PBMCs by cladribine *in vitro*. In contrast, gene and protein expression levels for NHLRC2 were significantly upregulated by the effect of cladribine (**Figure 4**). These four candidates were selected as potential biomarkers for *ex vivo* validation.

### 3.3 Identification of miRNA-gene interactions related to cladribine treatment

To select individual miRNAs involved in the regulation of the genes differentially expressed associated with cladribine exposure, we quantified the miRNAs using microarrays and further performed the differential expression analysis. The final miRNA dataset consisted of 6,350 probesets that included mature miRNAs, miRNA precursor stem-loops, snoRNA, HAcaBox, CDBox, scaRNA, and probesets targeting 5.8s rRNA. Fifty-five out of the 6,350 miRNAs were differentially expressed (p-values < 0.05 and absolute Log2Fold-Change > 0.5) between the cladribine treated and untreated conditions (**Figure 3B**). Compared to the untreated condition, 37 miRNAs were downregulated and 18 miRNAs upregulated after *in vitro* treatment with cladribine (**Figure 3B**). Next, analysis of the validated miRNA-target gene interactions led to the identification of 13,751 validated targets for the 55 dysregulated miRNAs (**Figure 3C**). Twenty-two out of these 55 miRNAs were known to regulate the four molecules that were differentially expressed both at the gene and protein levels between the cladribine treated and untreated conditions (**Table 2**). Five of the most dysregulated miRNAs were selected for *ex vivo* validation based on their biological and functional relevance to MS, as reported in the literature (**Table 2**).

**Table 2:** MiRNAs differentially expressed between the cladribine treated and untreated conditions that target selected genes.

miRNA	NIBAN2	NLHRC2	PPIF	JUN	Cladribine
**miR-484**	yes	yes			↓
let-7g-5p	yes	yes	yes		↓
miR-29c-3p	yes	yes		yes	↓
miR-15a-5p		yes		yes	↓
miR-8063		yes			↑
miR-29b-3p		yes		yes	↓
miR-192-5p		yes			↓
miR-26b-5p		yes		yes	↓
miR-29a-3p		yes		yes	↓
**miR-30b-5p**		yes		yes	↓
**miR-30e-5p**		yes		yes	↓
**miR-21-5p**			yes		↓
miR-27a-3p			yes		↓
miR-27b-3p			yes		↓
miR-128-3p			yes		↓
miR-106b-5p			yes		↓
miR-152-3p			yes		↓
miR-500a-3p			yes		↓
**miR-766-3p**				yes	↑
miR-532-5p				yes	↓
miR-99a-5p				yes	↓
miR-21-3p				yes	↓

↑ denotes upregulation of miRNA expression and ↓ denotes downregulation of miRNA expression by cladribine. “Yes” indicate that the gene is a target of the corresponding miRNA. In bold, miRNAs selected for ex vivo validation.

### 3.4 *Ex vivo* validation of cladribine effect in gene and miRNA expression of selected candidates

**Figure 5:**
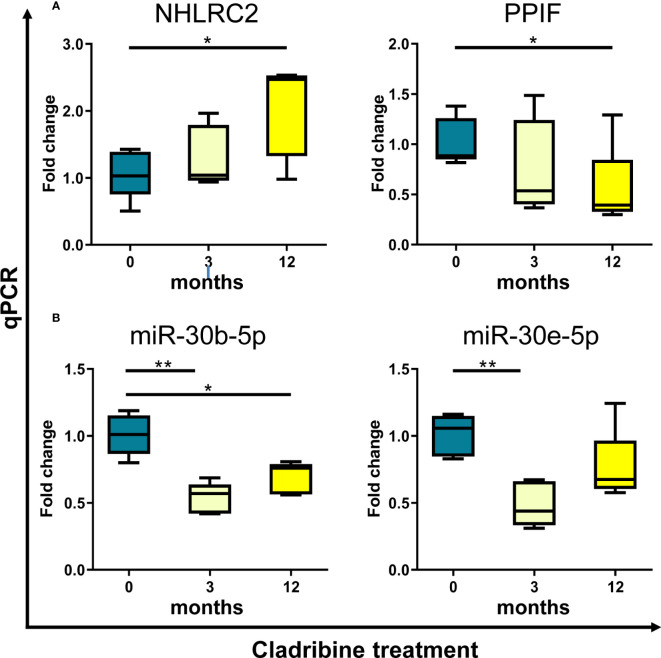
*Ex vivo* validation of selected candidate biomarkers by qPCR. Expression levels of the genes NHLRC2 and PPIF **(A)**, and the miRNAs miR-30b-5p and miR-30e-5p **(B)** were determined by qPCR in samples obtained from PBMCs of MS patients (N=5) treated with cladribine at baseline and after 3 and 12 months of treatment. Graphs are expressed as fold-change in gene and miRNA expression relative to baseline. *p-value <0.05, **p-value <0.01 (One-way ANOVA with repeated measures following a Dunnett multiple comparisons test, taking the baseline condition as reference). For the sake of clarity, only significant p-values are shown in the graphs. NHLRC2, NHL repeat containing 2. PPIF, Cyclophilin D.

As a final step, we approached the validation of the *in vitro* findings in PBMCs collected from MS patients treated with cladribine. For this *ex vivo* validation, the expression levels of the four selected molecules (NIBAN2, NHLRC2, PPIF, JUN) and the five selected miRNAs targeting these molecules (miR-484, miR-30b-5p, miR-30e-5p, miR-21-5p, miR-766-3p) were determined by qPCR at baseline and after 3 and 12 months of treatment. As depicted in **Figure 5A**, gene expression levels of NHLRC2 and PPIF increased and decreased respectively over time after cladribine treatment and differences reached statistical significance at 12 months compared to the baseline condition. In contrast, no significant differences were observed for NIBAN2 and JUN between the baseline and the different treated time points (**Supplementary Figure S2**). Interestingly, miR-30b-5p, a miRNA known to target NHLRC2, was significantly downregulated by the effect of treatment after 3 and 12 months (**Figure 5B**). Cladribine also significantly downregulated miR-30e-5p and miR-21-5p expression at 3 months, although expression levels for these two miRNAs were later increased at 12 months by the effect of treatment (**Figure 5B**; **Supplementary Figure S3**). No significant differences associated with cladribine treatment were observed for miR-766-3p and miR-484 (**Supplementary Figure S2**). To notice, expression levels for these selected candidates did not differ following stratification of patients into naïve and previously treated patients (data not shown).

## 4 Discussion

The goal of the present study was to gain insight into the molecular changes induced by cladribine treatment in MS patients. Using two multiomics approaches, we evaluated the whole transcriptome and proteome profiles, as well as the miRNAs that regulate their expression in samples obtained from PBMCs of MS patients treated *in vitro* with cladribine. In addition, a condition with cladribine in the presence of deoxycytidine was included to assess the effect of the drug in its inactive form. A first bioinformatics analysis integrating the three databases (gene/miRNA/protein) revealed a multiomics molecular signature induced by cladribine that was characterized by a general downregulation, particularly at the gene expression level. In addition, PBMCs treated with cladribine plus deoxycytidine showed an intermediate phenotype between the cladribine treated and the untreated cells at the three omics databases. A second bioinformatics approach analyzing each database separately and then merging the outcomes, confirmed the overall downregulating effect of cladribine identified in the first bioinformatics approach. Furthermore, this analysis also allowed to identify four candidate biomarkers, NIBAN2, NHLRC2, PPIF and JUN, and 22 miRNAs that regulated their expression, which were found dysregulated by the effect of cladribine. Data obtained *in vitro* were validated *ex vivo* in PBMCs of MS patients treated with cladribine for the genes NHLRC2 and PPIF, and for the miRNAs miR-30e-5p, miR-21-5p, and miR-30b-5p.

The cellular immunomodulatory effects of cladribine on PBMCs have been extensively described (8, 14). However, to present, the molecular effects associated with cladribine treatment remain elusive. Aiming to study the effect of cladribine in gene, protein and miRNA expression, PBMCs obtained from untreated MS patients were cultured, stimulated and then exposed to cladribine in the presence or absence of deoxycytidine. A recent study from our group demonstrated that cladribine showed an effect on PBMC activation after 4h of treatment in cells stimulated with PMA/ionomycin, and this effect was dependent and independent of DCK activity (8). In this context, PBMCs treated with cladribine plus deoxycytidine allowed to explore alternative pathways not requiring prodrug activation.

In order to obtain a more robust multiomics biomarker signature, stringent filtering criteria were applied, prior to integration, on each omics dataset to eliminate candidates that did not change in their expression levels with other members of the same dataset, or were not represented in most of the samples of the study. Then, we applied the DIABLO bioinformatics approach, a method that maximizes the common information between multiple omics datasets and finds coherent patterns associated to different phenotypes (23). The analysis clearly revealed that cladribine induced an overall downregulation mostly at the gene expression level. A comparable general downregulating effect at the transcriptional level has also been reported for other oral therapies like fingolimod in blood cells from MS patients (26). Interestingly, in our study PBMCs pretreated with deoxycytidine previous to the addition of cladribine showed an intermediate phenotype between cladribine treated cells and the untreated condition. These results suggest that inhibition of the activation of cladribine by DCK might induce different molecular pathways. In this regard, we and others have demonstrated that inhibition of cladribine activation induced changes at the cellular level (8, 27).

A crucial step in biomarker discovery is to narrow down the number of candidate targets from large omics datasets by identifying differentially regulated molecules (28). To achieve this goal, we applied the multi-stage approach focused to find differentially expressed features in each dataset and then merging the results. This approach allowed us to identify four common differentially expressed molecules, NIBAN2, NHLRC2, PPIF, and JUN, at the gene and protein levels. Although the multi-stage analysis found more than 200 genes and proteins dysregulated in PBMCs exposed to cladribine *in vitro* compared with untreated cells, the overlapping of the differentially expressed features identified only four candidates, which is most likely due to the stringent prefiltering steps performed before the analysis. However, other mechanisms controlling translation from mRNA to protein cannot be totally ruled out.

To validate the *in vitro* findings, we determined the levels of the four dysregulated genes in blood cells from MS patients receiving cladribine treatment. Microarray findings were validated *ex vivo* by qPCR for two of these candidates, NHLRC2 and PPIF, whose gene expression levels were significantly up- and downregulated respectively after 12 months of cladribine treatment. PPIF codes for cyclophilin D (CypD), a highly conserved peptidyl-prolyl cis-trans isomerase playing a crucial role in mitochondrial biology (29). CypD is a positive regulator of the mitochondrial permeability transition pore. The opening of this large and nonspecific conductance pore results into cell death following Ca2^+^ overload and ROS increase (30). In this line, mice treated with a CypD inhibitor recovered after induction of the MS animal model, experimental autoimmune encephalomyelitis (EAE), compared with untreated mice (31); and mice deficient for CypD developed EAE, but in contrast to wild type mice, they partially recovered and showed a striking axon preservation (32). In this context, the reduction in the CypD levels observed after cladribine treatment may be associated with the beneficial effects of cladribine in MS patients. Little is known in the literature about the function of the thioredoxin-like domain containing protein NHLRC2. Genetic variants in the NHLRC2 gene have been associated with the lethal FINCA (fibrosis, neurodegeneration, and cerebral angiomatosis) syndrome (33). NHLRC2 has also been proposed as a potent regulator of phagocytosis in human macrophages (34). Altered NHLRC2 protein and gene expression levels have been detected in neurodegenerative diseases such as Parkinson (35) and Alzheimer’s disease (36); however, publications on the role of NHLRC2 in MS are still lacking.

MiRNAs are small noncoding RNAs which are known to play critical roles in regulation of gene expression and protein production (37). They can act as negative or positive regulators of gene expression. To date, more than 13,000 experimentally validated miRNA–target interactions are available at different databases (24). Taking into account that miRNAs interact with a high number of targets, in our study the screening conditions for differential expression analysis to identify miRNAs were more restrictive than the conditions used for gene and protein selection. Twenty-two out of the 55 differentially expressed miRNA between cladribine treated and untreated cells presented an experimentally validated interaction with the four common differentially expressed genes-proteins identified in the same comparison, treated versus untreated. In order to examine the effect *ex vivo* of cladribine treatment in miRNA expression, we selected the top five differentially expressed miRNAs, including at least one interaction with each of the four candidates, and determined their expression levels by qPCR. Of these five, one miRNA, miR-30b-5p, was persistently downregulated during the first year of cladribine treatment, whereas two other miRNAs, miR-30e-5p and miR-21-5p, were only downregulated after 3 months of treatment but their expression was induced by the drug at 12 months. These data suggest that cladribine might have different timings in the regulation of the events involved in miRNA-mediated regulation of gene expression. Among the validated miRNAs, miR-21-5p is a prevalent miRNA across neurodegenerative diseases (38). Previous publications have demonstrated that miR-21-5p expression was increased during EAE, and its silencing alleviated the clinical signs (39, 40). In addition, miR-21-5p was upregulated in PBMCs of RRMS patients (41). Another miRNA whose levels were altered by cladribine is miR-30b-5p. This miRNA has been reported to be dysregulated in several neurodegenerative diseases with different functional roles (42). In particular, miR-30b-5p levels in serum exosomes helped to distinguish relapsing-remitting from progressive MS (43). Finally, the levels of miR-30e-5p were downregulated in the blood of patients with Parkinson disease compared with controls (44), but no reports on the role of miR-30e-5p have been found in MS.

Over the last years, “omics” technologies have emerged as a promising tool for identifying relevant molecular pathways and potentially attractive candidate biomarkers playing roles in the different disease phenotypes. However, many of these studies analyze genes, proteins and miRNAs independently through univariate statistical methods and ignore relationships between the different features that may contain crucial biological information. Hence, there is a growing desire to take more benefits of each omics technology through their integration. This field, known as multiomics, has the ability to discover novel disease mediators, candidate biomarkers, and new drug mechanisms of action missed by individual single omics studies. To our knowledge, this is the first study to integrate the gene-protein-miRNA expression profiles to better understand the underlying molecular mechanisms of a MS treatment.

In conclusion, by means of a combination of omics data and bioinformatics approaches we were able to identify not only a multiomics molecular profile consisting of genes, proteins and miRNAs associated with cladribine, but also a number of biomarkers whose expression levels were modified by cladribine in treated MS patients and have the potential to become treatment response biomarkers to this drug.

## Data availability statement

The datasets presented in this study can be found in online repositories. The names of the repository/repositories and accession number(s) can be found in the article/**Supplementary Material**.

## Ethics statement

The studies involving human participants were reviewed and approved by Vall d’Hebron Hospital, Barcelona [EPA(AG)57/2013(3834)] and the Hospital Ramón y Cajal, Madrid. The patients/participants provided their written informed consent to participate in this study.

## Author contributions

NF, and MC contributed to conception and design of study. NF, LC-B, HE, LV, LC-F, MF, AS, EB, ES, CE, XM, and MC contributed to the acquisition and analysis of data. NF, LC-B, HE, LV, LC-F, MF, AS, EB, ES, UB, CE, XM, and MC contributed to drafting a significant portion of the manuscript and figures. All authors contributed to the article and approved the submitted version.
