# Supplementary material for: Molecular signature associated with cladribine treatment in patients with multiple sclerosis
Source: Front Immunol. 2023 Jul 25;14:1233546. doi: 10.3389/fimmu.2023.1233546 (PMC10408299; doi:10.3389/fimmu.2023.1233546)
Supplement: Supplementary file 1 [file DataSheet_1.pdf]

Table S1: List of genes selected by the OMICS analysis and represented in the loading plots

Entrez	value.var	Affymetrix.ID	Gene.Symbol
1435	0.478684712	TC0100009364.hg.1	CSF1
57178	0.456459101	TC1000008182.hg.1	ZMIZ1
10215	0.345191257	TC2100006948.hg.1	OLIG2
64446	-0.291058409	TC1700008845.hg.1	DNAI2
11217	0.228936011	TC0900012157.hg.1	AKAP2
10184	0.199913828	TC0500011225.hg.1	LHFPL2
10512	0.180104809	TC0700011626.hg.1	SEMA3C
7067	0.172098143	TC1700007777.hg.1	THRA
165904	0.152071635	TC0300010772.hg.1	XIRP1
10257	0.146405443	TC1300009505.hg.1	ABCC4
444	0.144397912	TC0800010607.hg.1	ASPH
57182	0.135624604	TC0400011791.hg.1	ANKRD50
6793	0.129910159	TC0500012818.hg.1	STK10
51192	-0.118430998	TC1600011408.hg.1	CKLF
85406	0.115811303	TC1200012800.hg.1	DNAJC14
1879	0.109975405	TC0500012631.hg.1	EBF1
5176	-0.107361863	TC1700006524.hg.1	SERPINF1
23533	0.1036673	TC1700009708.hg.1	PIK3R5
586	0.094972391	TC1200010145.hg.1	BCAT1
1827	0.073212179	TC2100008030.hg.1	RCAN1
4327	0.063847356	TC1200010874.hg.1	MMP19
200734	0.0637274	TC0200012863.hg.1	SPRED2
51384	0.062453587	TC0700008930.hg.1	WNT16
6097	0.049229783	TC0100018491.hg.1	RORC
3082	0.046664223	TC0700011642.hg.1	HGF
8829	0.041066128	TC1000010273.hg.1	NRP1
6722	-0.040657997	TC0600008073.hg.1	SRF
9467	0.040260335	TC0300010399.hg.1	SH3BP5
123	0.035078644	TC0900012212.hg.1	PLIN2
272	0.034801966	TC1100012962.hg.1	AMPD3
284611	0.033775905	TC0100009324.hg.1	FAM102B
6446	0.026225931	TC0600013231.hg.1	SGK1
2012	0.021607218	TC1200006909.hg.1	EMP1
6347	0.019597783	TC1700007557.hg.1	CCL2
83985	0.015693664	TC1600011367.hg.1	SPNS1
116448	0.012968263	TC2100006951.hg.1	OLIG1
84669	0.011479718	TC1700011313.hg.1	USP32
10261	0.005331222	TC1600009683.hg.1	IGSF6
79760	-0.004461765	TC1900011764.hg.1	GEMIN7
6696	0.004248197	TC0400008091.hg.1	SPP1

[a]Entrez gene ID, [b]value.var.: variation of each feature in reference to the untreated group.

Table S2: List of proteins selected by the OMICS analysis and represented in the loading plots

Accession	value.var	Description
P38936	0.647292706	Cyclin-dependent kinase inhibitor 1
Q96I15	0.387750448	Selenocysteine lyase
Q96CV9	-0.278955259	Optineurin
P02787	-0.22174707	Serotransferrin
Q3MHD2	-0.219946069	Protein LSM12 homolog
P53990	0.216218487	IST1 homolog
P36405	-0.210136599	ADP-ribosylation factor-like protein 3
P25311	-0.201198657	Zinc-alpha-2-glycoprotein
P49792	0.190534159	E3 SUMO-protein ligase RanBP2
Q92918	0.180719619	Mitogen-activated protein kinase kinase kinase kinase 1
P02768	-0.149878	Serum albumin
Q14254	0.117674384	Flotillin-2
P02775	-0.104615506	Platelet basic protein
P02675	-0.063366876	Fibrinogen beta chain
P00738	-0.042850314	Haptoglobin
Q8N6H7	0.030494221	ADP-ribosylation factor GTPase-activating protein 2
P20700	0.018808144	Lamin-B1
Q96RQ9	0.017797303	L-amino-acid oxidase
P49790	0.015130461	Nuclear pore complex protein Nup153
P42330	0.003192348	Aldo-keto reductase family 1 member C3

[a]Accession number, [b]value.var.: variation of each feature in reference to the untreated group.

Table S3: List of miRNAs selected by the OMICS analysis and represented in the loading plots

Accession	value.var	ProbesetName	Symbol
MIMAT0000084	0.392599512	MIMAT0000084_st	hsa-miR-27a-3p
MIMAT0000069	0.300384723	MIMAT0000069_st	hsa-miR-16-5p
MIMAT0000440	-0.291111425	MIMAT0000440_st	hsa-miR-191-5p
MI0003684	0.275207376	MI0003684_st	hsa-mir-660
MIMAT0027629	-0.259652678	MIMAT0027629_st	hsa-miR-6864-3p
MIMAT0019764	0.234374994	MIMAT0019764_st	hsa-miR-4680-5p
MI0025753	0.225936891	MI0025753_st	hsa-mir-7977
MI0017281	0.224457552	MI0017281_st	hsa-mir-4653
MIMAT0000692	0.215815857	MIMAT0000692_st	hsa-miR-30e-5p
MIMAT0000510	-0.189059232	MIMAT0000510_st	hsa-miR-320a
MIMAT0000087	0.171800147	MIMAT0000087_st	hsa-miR-30a-5p
ENSG00000252050	-0.158474868	ENSG00000252050_st	ENSG00000252050
MI0003173	-0.158387127	MI0003173_x_st	hsa-mir-518a-2
MIMAT0004949	-0.155554955	MIMAT0004949_st	hsa-miR-877-5p
MIMAT0003888	-0.142758784	MIMAT0003888_st	hsa-miR-766-3p
MIMAT0016861	0.140590149	MIMAT0016861_st	hsa-miR-4308
ENSG00000238935	-0.135590186	ENSG00000238935_s_st	ENSG00000238935
MIMAT0000086	0.130087364	MIMAT0000086_st	hsa-miR-29a-3p
ENSG00000239038	-0.122179261	ENSG00000239038_x_st	ENSG00000239038
MIMAT0000068	0.117575278	MIMAT0000068_st	hsa-miR-15a-5p
MIMAT0022487	-0.110763489	MIMAT0022487_st	hsa-miR-5694
SNORD126	-0.106220614	SNORD126_st	SNORD126
MIMAT0027570	0.100715296	MIMAT0027570_st	hsa-miR-6835-5p
MIMAT0019785	-0.090003666	MIMAT0019785_st	hsa-miR-4693-3p
MI0015828	-0.087612316	MI0015828_st	hsa-mir-4301
MI0006318	-0.082736576	MI0006318_st	hsa-mir-1228
ENSG00000201619	-0.082723061	ENSG00000201619_st	ENSG00000201619
MIMAT0004748	-0.072955494	MIMAT0004748_st	hsa-miR-423-5p
MIMAT0000680	0.06757155	MIMAT0000680_st	hsa-miR-106b-5p
MIMAT0005929	-0.042643135	MIMAT0005929_st	hsa-miR-1275
ENSG00000238294	0.040300787	ENSG00000238294_st	ENSG00000238294
MIMAT0022472	-0.031086085	MIMAT0022472_st	hsa-miR-5683
MIMAT0005792	-0.030760327	MIMAT0005792_st	hsa-miR-320b
MIMAT0000073	0.029585586	MIMAT0000073_st	hsa-miR-19a-3p
MIMAT0005793	-0.026810323	MIMAT0005793_st	hsa-miR-320c
MI0006391	-0.022183805	MI0006391_st	hsa-mir-1257
MIMAT0022967	-0.017741807	MIMAT0022967_st	hsa-miR-3620-5p
ENSG00000239171	-0.007851521	ENSG00000239171_st	ENSG00000239171
MIMAT0018086	-0.005728448	MIMAT0018086_st	hsa-miR-3664-5p
MIMAT0005875	0.00572813	MIMAT0005875_st	hsa-miR-548j-5p

[a]Accession number, [b]value.var.: variation of each feature in reference to the untreated group. Probeset Name[c]

Figure S1

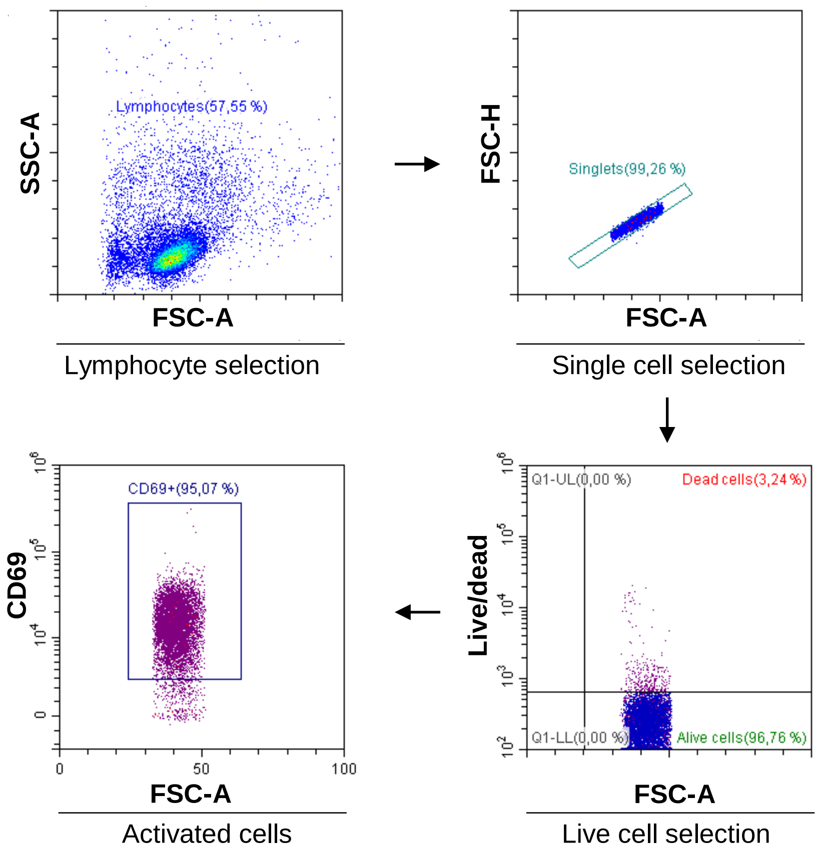

Figure S1: Activation status test by flow cytometry. Dot plots showing the gating strategy used to evaluate the activation of PBMCs. Lymphocytes were defined by SSC-Area and FSC-Area. Doublets (FSC-H and FSC-A gating), alive cells (fixable viability stain) were included in the analysis. Activation of PBMCs was analyzed by means of CD69 staining. A representative sample of PBMCs stimulated with PMA/ionomycin and simultaneously treated with 0.1 µM cladribine is shown.

Figure S2

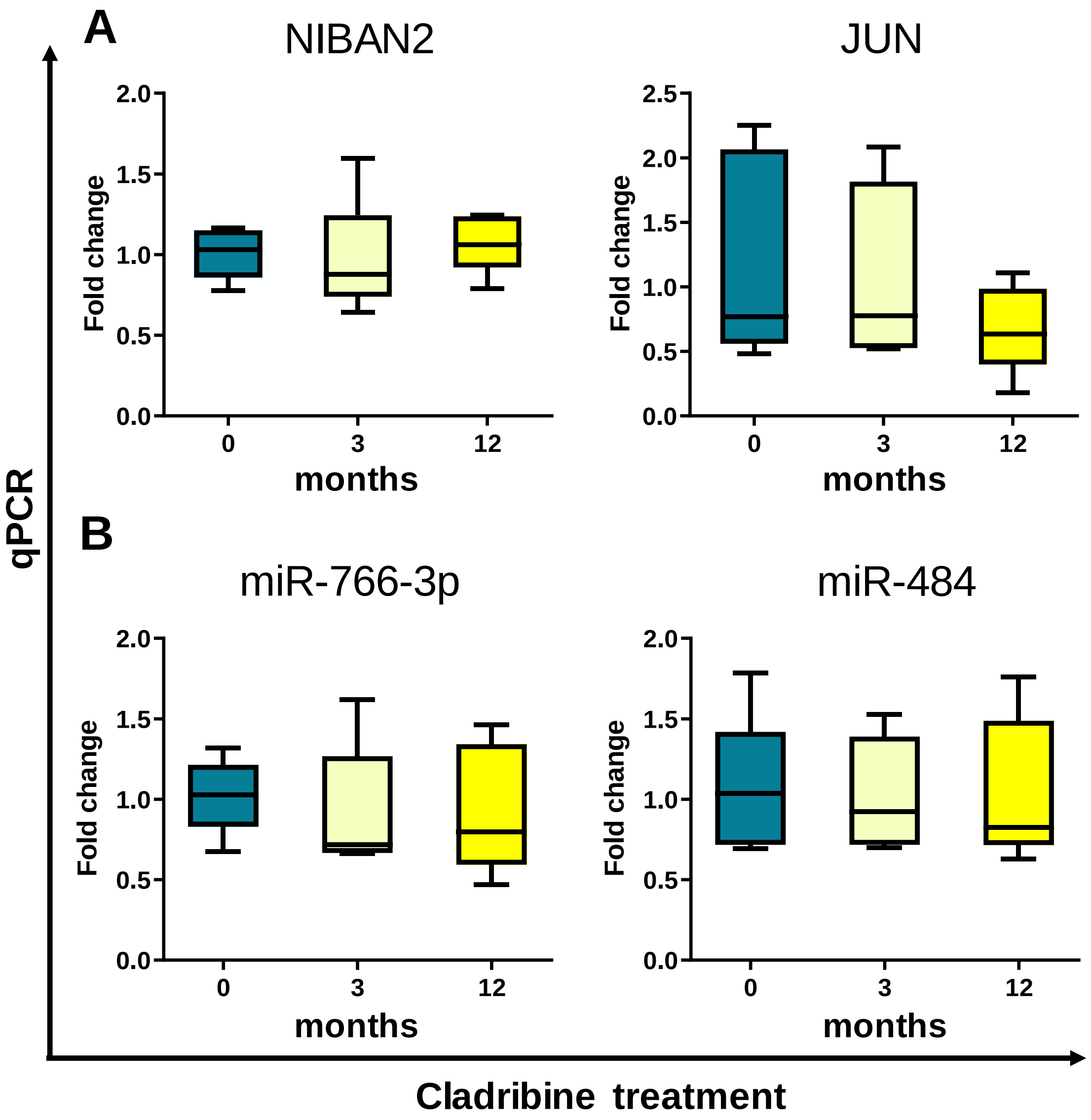

Figure S2: *Ex vivo* validation of selected candidate biomarkers by qPCR. Expression levels of the genes *NIBAN2* and *JUN* and the miRNAs *miR-766-3p* and *miR-484* were determined by qPCR in samples obtained from PBMCs of MS patients (N=5) treated with cladribine at baseline and after 3 and 12 months of treatment. Graphs are expressed as fold-change in gene and miRNA expression relative to baseline. NIBAN2: niban apoptosis regulator 2. JUN: Transcription factor Jun.

Figure S3

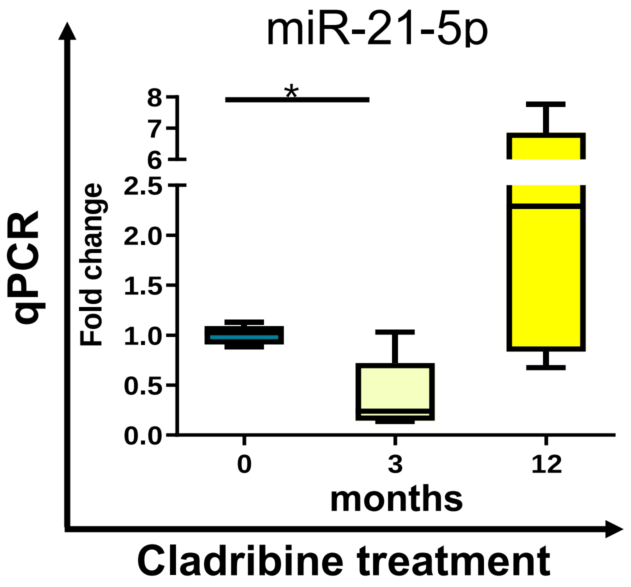

Figure S3: *Ex vivo* validation of *miR-21-5p* by qPCR. Expression levels of the miRNA miR-21-5p were determined by qPCR in samples obtained from PBMCs of MS patients (N=5) treated with cladribine at baseline and after 3 and 12 months of treatment. Graphs are expressed as fold-change in miRNA expression relative to baseline. *p-value <0.05, (One-way ANOVA with repeated measures following a Dunnett multiple comparisons test, taking the baseline condition as reference). A y-axis segmentation was performed to represent better high and low levels.

Supplementary Methods

Flow Cytometry

After *in vitro* stimulation with PMA/Ionomycin and simultaneous treatment with cladribine, $3x10^5$ PBMCs were harvested and stained for flow cytometry analysis in order to test cell activation by means of CD69 expression. PBMCs were stained using a fluorochrome-conjugated antibody against CD69 (Ref. 555533, BD Biosciences, San Jose, CA, USA). Discrimination of dead cells was achieved by Fixable Viability Stain (eBioscience, San Diego, CA, USA). Samples were acquired with a Cyto FLEX flow cytometer (Beckman Coulter, Brea, CA, USA) and data were analyzed with CytExpert 2.3 software (Beckman Coulter)
